# Prognostic role of neutrophil-to-lymphocyte ratio in breast cancer: a systematic review and meta-analysis

**DOI:** 10.1186/s13058-016-0794-1

**Published:** 2017-01-05

**Authors:** Josee-Lyne Ethier, Danielle Desautels, Arnoud Templeton, Prakesh S. Shah, Eitan Amir

**Affiliations:** 1 Division of Medical Oncology and Hematology, Princess Margaret Cancer Centre, 610 University Avenue 5-124, Toronto, ON M5G 2M9 Canada; 2 Department of Medicine, University of Toronto, Toronto, Canada; 3 Division of Medical Oncology and Hematology, Sunnybrook Health Sciences Centre, University of Toronto, Toronto, Canada; 4 Department of Medical Oncology, St. Claraspital Basel and Faculty of Medicine, University of Basel, Basel, Switzerland; 5 Institute of Health Policy and Management Evaluation, University of Toronto, Toronto, Canada; 6 Department of Paediatrics, Mount Sinai Hospital, University of Toronto, Toronto, Canada

**Keywords:** Breast cancer, Neutrophil-to-lymphocyte ratio, Prognosis, Disease-free survival, Overall survival, Meta-analysis, Systematic review

## Abstract

**Background:**

The presence of a high neutrophil-to-lymphocyte ratio (NLR) has been associated with increased mortality in several malignancies. Here, we quantify the effect of NLR on survival in patients with breast cancer, and examine the effect of clinicopathologic factors on its prognostic value.

**Methods:**

A systematic search of electronic databases was conducted to identify publications exploring the association of blood NLR (measured pre treatment) and overall survival (OS) and disease-free survival (DFS) among patients with breast cancer. Data from studies reporting a hazard ratio (HR) and 95% confidence interval (CI) or a *P* value were pooled in a meta-analysis. Pooled HRs were computed and weighted using generic inverse variance. Meta-regression was performed to evaluate the influence of clinicopathologic factors such as age, disease stage, tumor grade, nodal involvement, receptor status, and NLR cutoff on the HR for OS and DFS. All statistical tests were two-sided.

**Results:**

Fifteen studies comprising a total of 8563 patients were included. The studies used different cutoff values to classify high NLR (range 1.9–5.0). The median cutoff value for high NLR used in these studies was 3.0 amongst 13 studies reporting a HR for OS, and 2.5 in 10 studies reporting DFS outcomes. NLR greater than the cutoff value was associated with worse OS (HR 2.56, 95% CI = 1.96–3.35; *P* < 0.001) and DFS (HR 1.74, 95% CI = 1.47–2.07; *P* < 0.001). This association was similar in studies including only early-stage disease and those comprising patients with both early-stage and metastatic disease. Estrogen receptor (ER) and HER-2 appeared to modify the effect of NLR on DFS, because NLR had greater prognostic value for DFS in ER-negative and HER2-negative breast cancer. No subgroup showed an influence on the association between NLR and OS.

**Conclusions:**

High NLR is associated with an adverse OS and DFS in patients with breast cancer with a greater effect on disease-specific outcome in ER and HER2-negative disease. NLR is an easily accessible prognostic marker, and its addition to established risk prediction models warrants further investigation.

## Background

The short-term and long-term prognosis of breast cancer depends on patient and tumor factors such as age, disease stage, and biological factors such as grade and receptor status. However, the behavior of breast cancer is unpredictable, with markedly different clinical outcomes seen even amongst patients with similar classical prognostic factors [1].

Inflammatory cells and mediators in the tumor microenvironment are thought to play an important role in cancer progression, and may account for some of this variability [2]. The presence of an elevated peripheral neutrophil-to-lymphocyte (NLR) ratio, an indicator of systemic inflammation, has been recognized as a poor prognostic factor in various cancers [3]. In a previous meta-analysis of 100 studies of patients with unselected solid tumors, increased NLR was associated with decreased overall survival (OS) (hazard ratio (HR) 1.81; 95% confidence interval (CI) = 1.67–1.97; *P* < 0.001) [4]. This effect was observed in all disease sites, subgroups, and stages. However, this study was not specific to breast cancer, and did not examine the impact of prognostic factors such as estrogen receptor (ER) or progesterone receptor (PR) status, HER2 status, disease stage, or menopausal status.

The aim of this study was to quantify the effect of peripheral blood NLR on OS and disease-free survival (DFS) in adult women with invasive breast cancer. We also examined the effect of clinicopathologic factors on the prognostic value of NLR.

## Methods

### Data sources and searches

This analysis was reported in accordance with the Preferred Reporting Items for Systematic Reviews and Meta-Analyses (PRISMA) guidelines [5]. The search strategy developed by Templeton et al. [4] was used with the addition of “breast neoplasms” and synonymous breast cancer-specific terms. An electronic search of the following databases was performed: Medline (host: OVID), Medline in Process, Medline Epub Ahead of Print (host: OVID), EMBASE (host: OVID), and Cochrane Database of Systematic Reviews. All databases were searched from January 2013 to April 2016, supplementing the initial systematic review that searched databases until different time points in 2013. Citation lists of retrieved articles were screened manually to ensure sensitivity of the search strategy. The full search strategy is described in Table 3 in Appendix 1.

### Study selection

In order to reduce clinical heterogeneity, the following eligibility criteria were utilized: studies of adult women with breast cancer reporting on the prognostic impact of the peripheral blood NLR, where NLR was treated as a categorical variable; NLR collected prior to all treatment (surgery and/or systemic therapy); reporting of a multivariable HR for OS, and/or DFS or progression-free survival (PFS), and corresponding 95% CI and/or *P* value; available as a full-text publication; clinical trials, cohort studies, or case–control studies; and English-language publication. Case reports, conference proceedings, and letters to editors were excluded. Corresponding authors were contacted to clarify missing or ambiguous data. When multiple publications or data analyses were available from the same dataset and if clarification on potentially duplicate data could not be obtained, the study reporting the larger number of patients was retained and other studies were excluded. Studies only presenting data in graphic form without reporting a numerical value for HR were excluded. All titles identified by the search were evaluated, and all potentially relevant publications were retrieved in full. Two reviewers (JE and DD) independently reviewed full articles for eligibility based on inclusion criteria and data extraction, and disagreements were resolved by consensus. Three relevant articles identified in the previous systematic review were also included [4].

### Data extraction

The following details were extracted from included studies using predesigned data abstraction forms: name of first author, year of publication, journal, number of patients included in analysis, median age, disease stage (nonmetastatic, metastatic, mixed (nonmetastatic and metastatic)), collection of data (prospective, retrospective), cutoff value used to define high NLR, number of patients with each breast cancer subtype, number of premenopausal and postmenopausal patients, and HRs and associated 95% CIs for OS, PFS, or DFS. Where more than one multivariable model was reported, HRs were extracted from models including the most participants.

### Risk of bias assessment

Validity of included studies was assessed by two independent reviewers (J-LE and DD) using the Quality in Prognostic Studies (QUIPS) tool as described previously [6]. The QUIPS tool comprises 30 questions categorized into six domains (study participation, study attrition, prognostic factor measurement, outcome measurement, study confounding, and statistical analysis and reporting). Studies were rated according to each domain as being at low, moderate, or high risk of bias, based on the likelihood that they might alter the relationship between the prognostic factor and outcome.

### Statistical analyses

Extracted data were pooled using RevMan 5.3 analysis software (Cochrane Collaboration, Copenhagen, Denmark). A meta-analysis was conducted for all included studies for each of the endpoints of interest if appropriate when clinical heterogeneity was minimal. The primary outcome of interest was OS, and intermediate endpoints such as PFS and/or DFS were secondary outcomes. Estimates for HRs were pooled and weighted by generic inverse variance, and were computed by fixed-effects or random-effects modeling. Heterogeneity was assessed using Cochran *Q* and *I* ^2^ statistics. If significant heterogeneity was present (*I* ^2^ > 50% or Cochran *Q* < 0.1), a random-effects model was used. Predefined subgroup analyses were conducted for disease stage (early, metastatic, mixed) using methods described by Deeks et al. [7] Meta-regression was performed to evaluate the effects of NLR cutoff, proportion of ER-positive patients, proportion of HER2-positive patients, proportion of triple-negative patients, median age, proportion of premenopausal patients, and proportion of patients with metastatic disease on the HR for OS and DFS. Meta-regression comprised a univariable linear regression weighted by individual study inverse variance and was performed using SPSS version 24 (IBM Corp, Armonk, NY, USA). A post-hoc meta-regression analysis testing the association between median duration of follow-up and the prognostic value of NLR was also performed. Multivariable meta-regression was not performed due to the small number of eligible studies leading to an undesirable risk of over-fitting. Publication bias was assessed by inspecting funnel plots visually. All statistical tests were two-sided, and statistical significance was defined as *P <* 0.05.

## Results

**Fig. 1:**
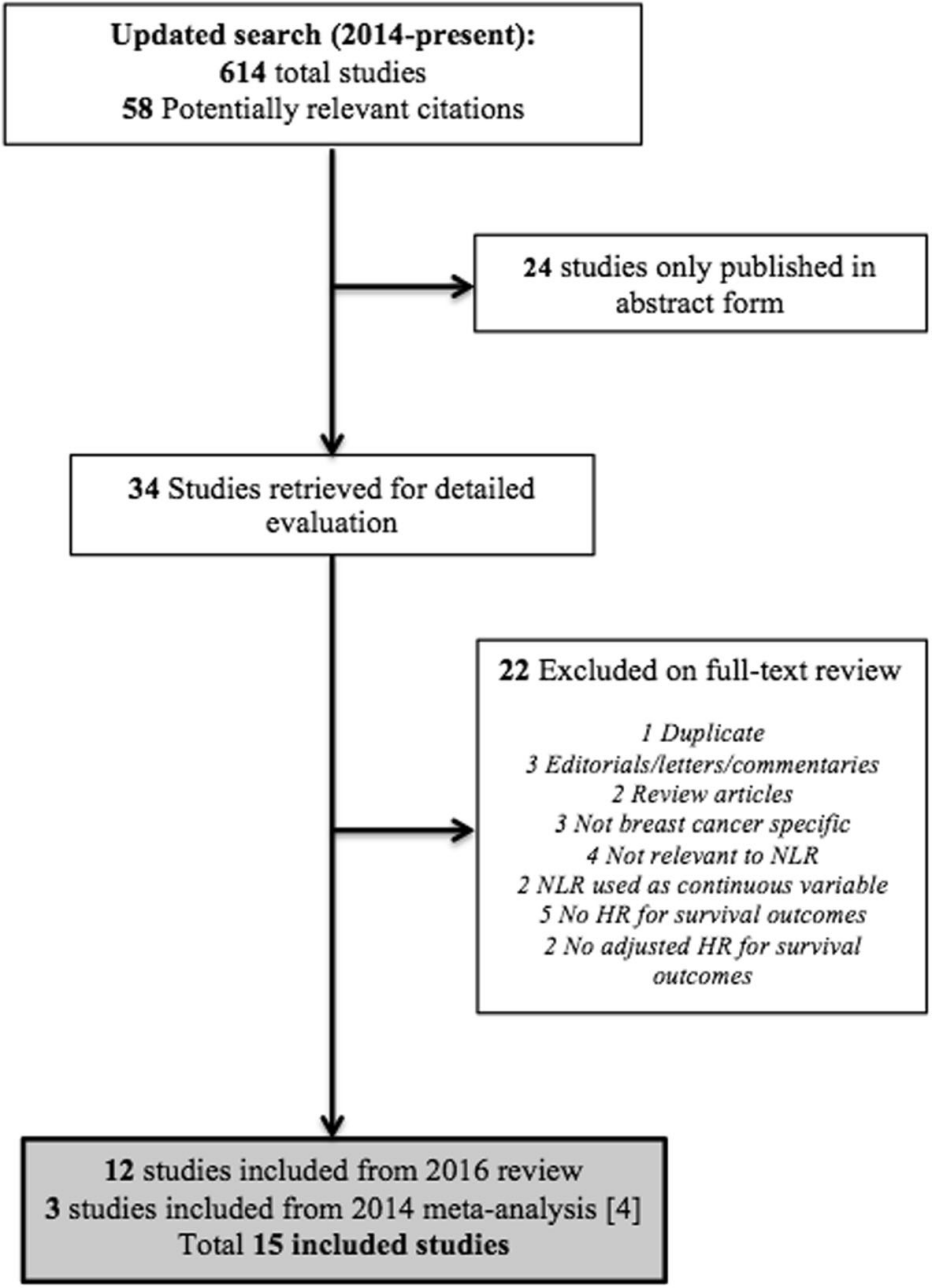
Flow chart of study selection process. *HR* hazard ratio, *NLR* neutrophil-to-lymphocyte

Fifteen studies comprising a total of 8563 patients were included (Fig. 1). Characteristics of included studies are described in Table 1, and further details are included in Table 4 in Appendix 2. All studies collected data retrospectively, and all were published in 2012 or later. Ten studies included only patients with early-stage breast cancer, while five included both early and metastatic disease.

**Table 1:** Characteristics of included studies

Study	Year	Number of patients	Disease stage	NLR cutoff value
Overall survival				
Azab et al. [23]^a^	2012	316	Mixed	3.3
Azab et al. [13]^a^	2013	437	Mixed	3.3
Bozkurt et al. [24]	2015	85	Early	2.0
Dirican et al. [25]	2015	1527	Mixed	4.0
Forget et al. [10]	2014	720	Early	3.3
Jia et al. [14]	2015	1570	Early	2.0
Koh et al. [8]	2014	157	Early	2.3
Koh et al. [15]	2015	1435	Mixed	5.0
Nakano et al. [9]	2015	167	Early	2.5
Noh et al. [26]^a^	2013	442	Early	2.5
Pistelli et al. [27]	2015	90	Early	3.0
Rimando et al. [28]	2016	461	Mixed	3.8
Yao et al. [11]	2014	608	Early	2.6
Disease-free survival				
Asano et al. [12]	2016	61	Early	3.0
Bozkurt et al. [24]	2015	85	Early	2.0
Dirican et al. [25]	2015	1527	Mixed	4.0
Forget et al. [10]	2014	720	Early	3.3
Hong et al. [29]	2015	487	Early	1.9
Jia et al. [14]	2015	1570	Early	2.0
Koh et al. [8]	2014	157	Early	2.3
Nakano et al. [9]	2015	167	Early	2.5
Pistelli et al. [27]	2015	90	Early	3.0

*NLR* neutrophil-to-lymphocyte

^a^Included in previous meta-analysis [4]

### Overall survival

**Fig. 2:**
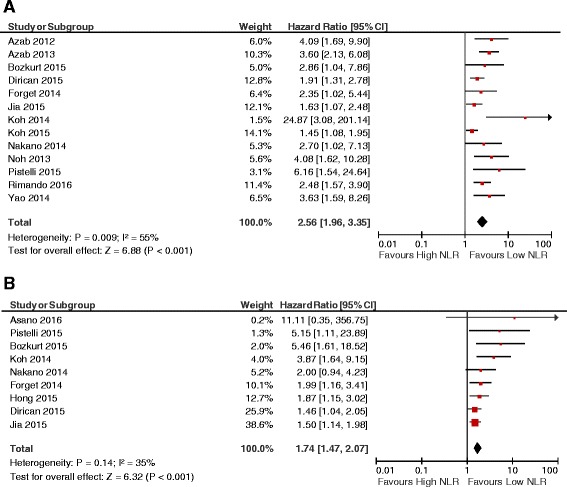
Forest plots showing HRs for OS (**a**) and DFS (**b**) for neutrophil-to-lymphocyte ratio (*NLR*) greater than or less than the cutoff value. HRs for each study represented by *squares*: *size* of the square represents the weight of the study in the meta-analysis, and the *horizontal line* crossing the square represents the 95% confidence interval (*CI*). All statistical tests were two-sided

Thirteen studies comprising a total of 8015 patients reported adjusted HRs for OS. The median cutoff value for high NLR was 3.0 (range 2.0–5.0). Median follow-up was reported in 11 studies, and ranged from 1.8 to 7.2 years (mean 4.69 years) (Table 4 in Appendix 2). Overall, a NLR greater than the cutoff value was associated with worse OS (HR 2.56, 95% CI = 1.96–3.35; *P* < 0.001; see Fig. 2). There was statistically significant heterogeneity (Cochran *Q* = 0.009, *I* ^2^ = 55%). This seems to be largely influenced by one study which showed a large effect size [8]. However, the association between NLR and OS was maintained in a sensitivity analysis omitting this study (HR 2.42, 95% CI = 1.89–3.09; *P* < 0.001; Cochran *Q* = 0.03, *I* ^2^ = 48%), although statistically significant heterogeneity remained.

Exploratory analysis identified breast cancer stage as an important source of heterogeneity. Subgroup analysis showed that the association between NLR and OS was maintained in studies including only early-stage disease, as well as those comprised of patients with both early and metastatic disease (HR 2.98 vs 2.30 respectively; *P* for subgroup differences = 0.36). There was no statistical heterogeneity when the study driving heterogeneity in the main analysis [8] was omitted from the early stage subgroup (Cochran *Q* = 0.28, *I* ^2^ = 20%). Additionally, the effect of NLR on OS was retained (HR 2.56, 95% CI = 1.82–3.60; *P* < 0.001). Statistical heterogeneity remained among studies with mixed early and metastatic disease (Cochran *Q* = 0.01, *I* ^2^ = 69%).

Adjustment for age differences between arms was examined in individual studies. In one study, patients were significantly older in the arm with low NLR, and it was unclear whether the multivariable model was adjusted for age [9]. In two other studies, the median age in each arm was not reported, and age did not seem to be included in the multivariable model [10, 11]. In a sensitivity analysis excluding these three studies, high NLR remained a significant predictor for shorter OS (HR 2.55, 95% CI = 2.59–8.26; *P* < 0.001). Table 2 presents the results of the meta-regression analysis. We did not identify any classical clinicopathologic factors that were effect modifiers for influence of NLR on OS. Additionally, the median duration of follow-up did not affect the association between high NLR and OS.

**Table 2:** Meta-regression for the association of clinicopathologic factors and the hazard ratio for disease-free and overall survival

Variable	Studies included in analysis	Standardized β coefficient	*P* value
Overall survival			
Median age	[8, 9, 11, 13–15, 26–28]	0.098	0.80
ER positive	[9–11, 13, 15, 23–27]	0.084	0.81
HER2 positive	[8–11, 14, 15, 23–27]	–0.40	0.22
Triple negative	[8, 14, 24, 27]	0.05	0.93
Grade 1 or 2	[8, 10, 14, 15, 23–25]	0.02	0.95
Grade 3	[8, 10, 14, 15, 23–25]	–0.02	0.95
Stage 0–I	[9, 13, 23, 25, 27, 28]	0.68	0.14
Stage II	[9, 13, 23, 25, 27, 28]	–0.30	0.56
Stage III	[9, 13, 25, 27, 28]	–0.73	0.16
Metastatic disease	[8–11, 13–15, 24–28]	–0.29	0.35
Premenopausal	[24, 25]	0.04	0.95
Nodal involvement	[8–11, 13–15, 23–27]	–0.04	0.90
NLR cutoff value	[8, 10, 13–15, 23, 24]	–0.29	0.33
Median follow-up	[8–11, 13, 14, 23, 25–28]	–0.16	0.64
Disease-free survival			
Median age	[8, 9, 14, 27, 29]	0.06	0.93
ER positive	[9, 10, 12, 24, 25, 27, 29]	–0.77	0.04*
HER2 positive	[8–10, 12, 14, 24, 25, 27, 29]	–0.79	0.01*
Triple negative	[8, 12, 14, 24, 27, 29]	0.63	0.18
Grade 1 or 2	[8–10, 12, 14, 24, 25, 27, 29]	–0.46	0.21
Grade 3	[8–10, 12, 14, 24, 25, 27, 29]	0.46	0.21
Stage 0–I	[9, 25, 27, 29]	0.46	0.54
Stage II	[9, 25, 27, 29]	0.53	0.36
Stage III	[9, 25, 27, 29]	–0.50	0.39
Metastatic disease	[25]	–0.74	0.49
Premenopausal	[9, 12, 24, 25, 27]	0.43	0.40
Nodal involvement	[8–10, 12, 14, 24, 25, 27, 29]	0.25	0.52
NLR cutoff value	[8–10, 12, 14, 24, 25, 27, 29]	–0.15	0.70
Median follow-up	[8–10, 12, 14, 25, 27, 29]	–0.19	0.66

*ER* estrogen receptor, *NLR* neutrophil-to-lymphocyte

*Statistically significant at *P <* 0.05

**Fig. 3:**
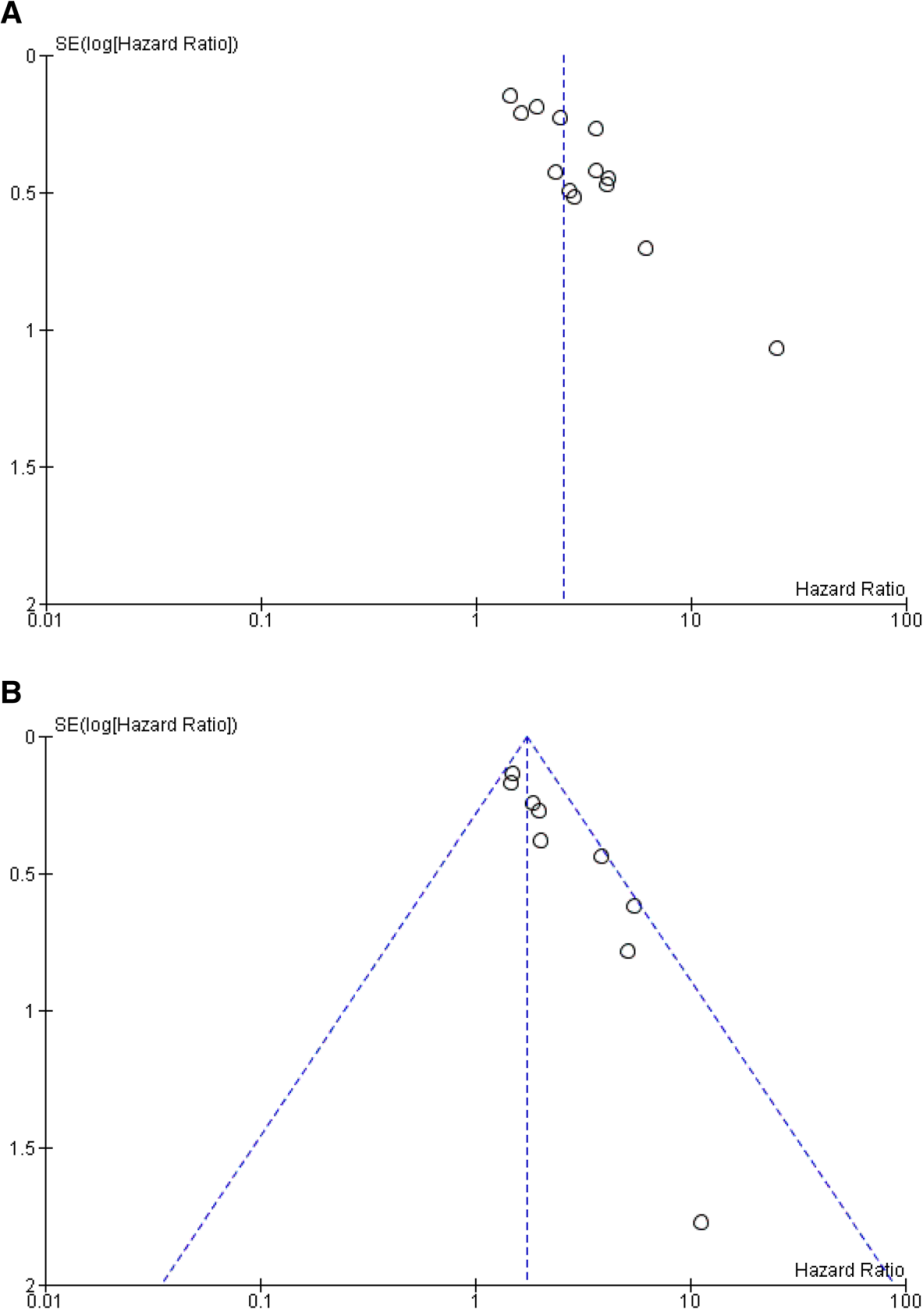
Funnel plots of HR for OS (**a**) and DFS (**b**) for high NLR ratio (*horizontal axis*) and the standard error (*SE*) for the HR (*vertical axis*). Each study is represented by one *circle. Vertical line* represents the pooled effect estimate

There was evidence of publication bias, with fewer smaller studies reporting lower magnitude associations between NLR and OS (Fig. 3).

### Disease-free survival

Nine studies comprising 4864 patients reported HRs for DFS. All studies included only patients with nonmetastatic disease. The median cutoff value for high NLR was 2.5 (range 1.9–4.0). Median length of follow-up was reported in eight studies, ranging from 1.8 to 7.2 years (mean 4.5 years) (Table 4 in Appendix 2). Overall, a NLR greater than the cutoff value was associated with worse DFS (HR 1.74, 95% CI = 1.47–2.07; *P* < 0.001; see Fig. 2). There was no evidence of statistically significant heterogeneity (Cochran *Q* = 0.14, *I* ^2^ = 35%).

Adjustment for age differences between arms was examined in individual studies. Two studies had significant age differences between arms and no clear model adjustment for age, including one study where patients were significantly older in the arm with low NLR [9] and one study where the same group was significantly younger [12]. Another study did not report the median age in each arm and did not adjust for age in the multivariable model [10]. In a sensitivity analysis excluding these three studies, high NLR remained a significant predictor for shorter DFS (HR 1.69, 95% CI = 1.40–2.03; *P* < 0.001).

All studies reported the number of patients with HER2-positive disease, while seven of nine studies included data on ER status (Table 4 in Appendix 2). Meta-regression analysis is presented in Table 2. Results showed that ER and HER2 positivity were negative effect modifiers of the association between NLR and DFS, indicating that the NLR has a greater prognostic value in breast cancers that are ER-negative and/or HER2-negative. The proportion of patients with triple-negative or metastatic disease, median age, disease stage, histologic tumor grade, presence of nodal involvement, premenopausal status, median duration of follow-up, and NLR cutoff value did not affect the association between high NLR and DFS. There was evidence of publication bias, with fewer smaller studies reporting lower magnitude associations between NLR and DFS (Fig. 3).

### Risk of bias assessment

**Fig. 4:**
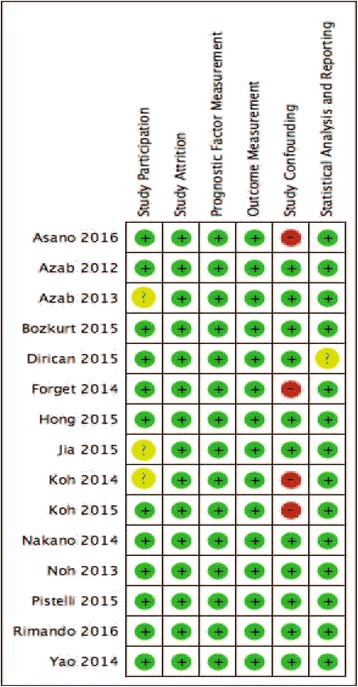
Risk of bias summary: review authors’ judgments using the Quality in Prognostic Studies (QUIPS) tool [6]. Domains are rated as being at low (*+*), moderate (*?*), or high (*–*) risk of bias

The risk of bias in individual studies is summarized in Figure 4 in Appendix 3. Overall, risk of bias was low, particularly in the domains of study attrition, prognostic factor measurement, outcome measurement, and statistical analysis and reporting. There was a low–moderate risk of bias for the study participation domain due to lack of completeness in description of the baseline study sample in three studies [8, 13, 14]. Risk of bias was moderate with regards to study confounding, because four studies failed to adequately detail covariates included in adjusted models [8, 10, 12, 15].

## Discussion

High NLR is associated with poor survival in patients diagnosed with several types of cancer [4]. Here we performed a breast cancer-specific meta-analysis, including 15 studies comprising 8563 patients, and found a significant prognostic effect for NLR on both OS and DFS. While there was evidence of publication bias, potentially indicating bias towards publication of positive studies, the overall risk of bias was low, as assessed with the QUIPS tool.

The magnitude of effect on DFS was highest in ER-negative and HER2-negative subtypes. However, this finding does not rule out an effect in ER-positive or HER2-positive subgroups. Rather, the finding indicates a greater magnitude of effect in ER-negative and/or HER2-negative breast cancers. It is possible that the smaller magnitude of effect seen in ER-positive and/or HER2-positive disease relates to the relatively short duration of follow-up of included studies; recurrences occur later in follow-up with ER-positive disease compared with ER-negative disease. However, in a post-hoc meta-regression analysis, median follow-up did not significantly alter the association of NLR with either DFS or OS. Unfortunately, a stratified meta-regression based on ER status was not possible. Some uncertainty therefore remains about the effect of duration of follow-up on subgroups defined by receptor expression.

Despite a greater magnitude of association between NLR and DFS in certain subgroups, patient and disease characteristics did not significantly alter the magnitude of effect of NLR on OS. The negative prognostic effect of NLR on OS was consistent in all clinicopathologic groups and was not influenced by the duration of follow-up in individual studies. One possible explanation for this is that a proportion of breast cancer patients die of causes other than breast cancer, especially cardiovascular disease [16, 17]. Increased NLR has been associated with higher coronary heart disease mortality [18]. The competing risks of cardiovascular and breast cancer deaths may have led to difficulty in exploring the influence of breast cancer-specific characteristics on OS.

While the association between increased NLR and poor outcomes is not fully understood, it has been proposed that high NLR may be indicative of inflammation. In particular, neutrophils have been shown to inhibit the immune system and promote tumor growth by suppressing the activity of lymphocytes and T-cell response [19, 20]. Increased lymphocytic tumor infiltration has also been associated with improved DFS in ER-negative/HER2-negative breast cancer [21]. In our study, we found a greater magnitude of effect on DFS in patients with ER-negative and/or HER2-negative disease. However, while this indicates the potential importance of lymphocyte activity, the association between increased tumor-infiltrating lymphocytes and peripheral blood lymphocytes remains unclear. Furthermore, the greater magnitude of association in patients with ER-negative and/or HER2-negative breast cancers was not seen with triple-negative disease. This observation may be due to the relatively small number of studies reporting outcomes in patients with triple-negative breast cancer; the majority of studies identified patients based on independent subgroups based on ER and HER2 status.

While there are several clinicopathologic factors associated with increased risk of recurrence and/or mortality in patients with breast cancer, the NLR is an inexpensive, readily available prognostic marker, and may allow refinement of risk estimates within disease stages and subgroups. Future studies using NLR in combination with other prognostic markers could potentially identify lower risk patients in whom treatment de-escalation may be appropriate. Furthermore, whether NLR is predictive of response to treatment or provides additional information in cases where risk stratification models exist, such as the 21-gene assay in node-negative ER-positive/HER2-negative disease, is unknown. However, previous research showed no association between NLR and the 21-gene assay recurrence score, indicating that the poor outcomes in patients with high NLR cannot be explained by the proliferation of ER signaling [22]. Further studies examining whether NLR may help refine established prognostic scores are therefore warranted.

## Conclusion

High NLR is associated with an adverse OS and DFS in patients with breast cancer, and its prognostic value is consistent among different clinicopathologic factors such as disease stage and subtype. NLR is an easily accessible prognostic marker, and its addition to established risk prediction models warrants further investigation.

## Appendix 1

**Table 3:** Search strategy^a^

Number	Searches	Results	Type
1	exp Breast Neoplasms/	241,242	Advanced
2	(breast? adj6 cancer*).mp,kw.	203,097	Advanced
3	(breast? adj6 neoplas*).mp,kw.	241,382	Advanced
4	(breast? adj6 carcin*).mp,kw.	62,218	Advanced
5	(breast? adj6 tumo?r*).mp,kw.	46,556	Advanced
6	(breast? adj6 adenocarcin*).mp,kw.	4642	Advanced
7	(breast? adj6 adeno-carcin*).mp,kw.	10	Advanced
8	(breast? adj6 sarcoma*).mp,kw.	1271	Advanced
9	(breast? adj6 dcis).mp,kw.	1258	Advanced
10	(breast? adj6 ductal).mp,kw.	16,064	Advanced
11	(breast? adj6 infiltrating).mp,kw.	1418	Advanced
12	(breast? adj6 intraductal).mp,kw.	2294	Advanced
13	(breast? adj6 lobular).mp,kw.	4044	Advanced
14	(breast? adj6 medullary).mp,kw.	383	Advanced
15	(breast? adj6 comedo*).mp,kw.	75	Advanced
16	(breast? adj6 metast*).mp,kw.	26,054	Advanced
17	(breast? adj2 malignan*).mp,kw.	4962	Advanced
18	(breast? adj6 onco*).mp,kw.	3338	Advanced
19	(mammar* adj6 cancer*).mp,kw.	5493	Advanced
20	(mammar* adj6 neoplas*).mp,kw.	21,985	Advanced
21	(mammar* adj6 carcin*).mp,kw.	11,584	Advanced
22	(mammar* adj6 tumo?r*).mp,kw.	18,026	Advanced
23	(mammar* adj6 adenocarcin*).mp,kw.	2958	Advanced
24	(mammar* adj6 adeno-carcin*).mp,kw.	3	Advanced
25	(mammar* adj6 sarcoma*).mp,kw.	384	Advanced
26	(mammar* adj6 ductal).mp,kw.	937	Advanced
27	(mammar* adj6 intraductal).mp,kw.	117	Advanced
28	(mammar* adj6 infiltrating).mp,kw.	201	Advanced
29	(mammar* adj6 lobular).mp,kw.	151	Advanced
30	(mammar* adj6 medullary).mp,kw.	19	Advanced
31	(mammar* adj6 comedo*).mp,kw.	6	Advanced
32	(mammar* adj6 metast*).mp,kw.	2554	Advanced
33	(mammar* adj6 malignan*).mp,kw.	1506	Advanced
34	(mammar* adj6 dcis).mp,kw.	61	Advanced
35	(ductal adj6 situ).mp,kw.	6301	Advanced
36	(ductal adj6 carcino*).mp,kw.	25,790	Advanced
37	(paget?? adj6 breast?).mp,kw.	367	Advanced
38	(paget?? adj6 nipple?).mp,kw.	363	Advanced
39	phyllodes.mp,kw.	1876	Advanced
40	phylloides.mp,kw.	206	Advanced
41	cystosarcoma*.mp,kw.	603	Advanced
42	DCIS.mp,kw.	3401	Advanced
43	or/1-40	318,397	Advanced
44	exp Ovarian Neoplasms/	71,707	Advanced
45	(ovar* adj6 cancer*).mp,kw.	44,037	Advanced
46	(ovar* adj6 neoplas*).mp,kw.	71,929	Advanced
47	(ovar* adj6 tumo?r*).mp,kw.	24,113	Advanced
48	(ovar* adj6 malignan*).mp,kw.	7601	Advanced
49	(ovar* adj6 metasta*).mp,kw.	5781	Advanced
50	(ovar* adj6 carcin*).mp,kw.	18,742	Advanced
51	(ovar* adj6 adenocarcin*).mp,kw.	2966	Advanced
52	(ovar* adj6 adeno-carcin*).mp,kw.	12	Advanced
53	(ovar* adj6 choriocarcin*).mp,kw.	217	Advanced
54	(granulosa adj6 cancer*).mp,kw.	54	Advanced
55	(granulosa adj6 tumo?r*).mp,kw.	2699	Advanced
56	(granulosa adj6 neoplas*).mp,kw.	173	Advanced
57	(granulosa adj6 malignan*).mp,kw.	142	Advanced
58	(granulosa adj6 metasta*).mp,kw.	111	Advanced
59	(granulosa adj6 carcin*).mp,kw.	118	Advanced
60	(granulosa adj6 adenocarcin*).mp,kw.	45	Advanced
61	(granulosa adj6 adeno-carcin*).mp,kw.	0	Advanced
62	OGCTs.mp,kw.	28	Advanced
63	HBOC.mp,kw.	650	Advanced
64	Luteoma*.mp,kw.	203	Advanced
65	Sertoli-Leydig*.mp,kw.	1039	Advanced
66	Thecoma*.mp,kw.	1013	Advanced
67	(theca* adj6 tumo?r*).mp,kw.	493	Advanced
68	(ovar* adj6 dysgerminoma?).mp,kw.	467	Advanced
69	androblastoma*.mp,kw.	321	Advanced
70	arrhenoblastoma*.mp,kw.	349	Advanced
71	arrheno-blastoma*.mp,kw.	1	Advanced
72	Meig*.mp,kw.	2152	Advanced
73	or/44-72	93,590	Advanced
74	exp Endometrial Neoplasms/	17,416	Advanced
75	(endometr* adj6 neoplas*).mp,kw.	17,866	Advanced
76	(endometr* adj6 cancer*).mp,kw.	15,307	Advanced
77	(endometr* adj6 tumo?r*).mp,kw.	5128	Advanced
78	(endometr* adj6 carcino*).mp,kw.	12,730	Advanced
79	(endometr* adj6 adenocarcin*).mp,kw.	5361	Advanced
80	(endometr* adj6 adeno-carcin*).mp,kw.	9	Advanced
81	(endometr* adj6 sarcoma*).mp,kw.	1230	Advanced
82	(endometr* adj6 malignan*).mp,kw.	2300	Advanced
83	(endometr* adj6 metast*).mp,kw.	1337	Advanced
84	(endometr* adj6 onco*).mp,kw.	370	Advanced
85	(endometr* adj6 choriocarcin*).mp,kw.	88	Advanced
86	or/74-85	31,774	Advanced
87	Uterine Cervical Neoplasms/	65,130	Advanced
88	(cervi* adj6 cancer*).mp,kw.	41,277	Advanced
89	(cervi* adj6 neoplas*).mp,kw.	69,153	Advanced
90	(cervi* adj6 tumo?r*).mp,kw.	7715	Advanced
91	(cervi* adj6 malignan*).mp,kw.	3006	Advanced
92	(cervi* adj6 metast*).mp,kw.	6612	Advanced
93	(cervi* adj6 onco*).mp,kw.	1280	Advanced
94	(cervi* adj6 carcin*).mp,kw.	24,588	Advanced
95	(cervi* adj6 adenocarcin*).mp,kw.	2945	Advanced
96	(cervi* adj6 adeno-carcin*).mp,kw.	9	Advanced
97	(cervi* adj6 squamous*).mp,kw.	7833	Advanced
98	(cervi* adj6 adenosquamous*).mp,kw.	211	Advanced
99	(cervi* adj6 adeno-squamous*).mp,kw.	2	Advanced
100	(cervi* adj6 sarcoma*).mp,kw.	661	Advanced
101	(cervi* adj6 small cell*).mp,kw.	364	Advanced
102	(cervi* adj6 large cell*).mp,kw.	78	Advanced
103	(cervi* adj6 neuroendocrine*).mp,kw.	195	Advanced
104	(cervi* adj6 neuro-endocrine*).mp,kw.	2	Advanced
105	(cervi* adj6 choriocarcin*).mp,kw.	112	Advanced
106	SCCC.mp,kw.	46	Advanced
107	or/87-106	90,890	Advanced
108	73 or 86 or 107	199,155	Advanced
109	exp Lymphocytes/	461,529	Advanced
110	lymphocyte?.mp,kw.	554,948	Advanced
111	(lymphoid adj2 cell?).mp,kw.	22,666	Advanced
112	(killer adj4 cell?).mp,kw.	51,337	Advanced
113	(nk adj2 cell?).mp,kw.	31,413	Advanced
114	(lak adj2 cell?).mp,kw.	2650	Advanced
115	b-lymphocyte?.mp,kw.	93,264	Advanced
116	t-lymphocyte?.mp,kw.	290,882	Advanced
117	b-lymphoid.mp,kw.	2219	Advanced
118	t-lymphoid.mp,kw.	1196	Advanced
119	(plasm adj2 cell?).mp,kw.	31	Advanced
120	plasmacyte?.mp,kw.	341	Advanced
121	(immune adj3 cell?).mp,kw.	58,743	Advanced
122	(immunocompetent adj2 cell?).mp,kw.	3494	Advanced
123	immnunocyte?.mp,kw.	0	Advanced
124	immnuno-cyte?.mp,kw.	0	Advanced
125	lymph cell?.mp,kw.	184	Advanced
126	null cell?.mp,kw.	3404	Advanced
127	immunological* competent cell?.mp,kw.	153	Advanced
128	immunoreactive cell?.mp,kw.	6231	Advanced
129	immuno-reactive cell?.mp,kw.	18	Advanced
130	prolymphocyte?.mp.	218	Advanced
131	pro-lymphocyte?.mp.	3	Advanced
132	or/109-131	648,538	Advanced
133	Neutrophils/	77,202	Advanced
134	neutrophil*.mp,kw.	135,327	Advanced
135	(cell? adj2 le).mp,kw.	868	Advanced
136	(leukocyte? adj3 polymorphonuclear).mp,kw.	14,471	Advanced
137	pmn granulocyte?.mp,kw.	52	Advanced
138	pmn leukocyte?.mp,kw.	400	Advanced
139	(poly morphou* adj2 granulocyte?).mp,kw.	0	Advanced
140	(polynuclear adj3 leukocyte?).mp,kw.	71	Advanced
141	or/133-140	139,999	Advanced
142	(neutrophil? adj6 lymphocyte?).mp,kw.	8790	Advanced
143	NLR.mp,kw.	1729	Advanced
144	132 and 141	26,722	Advanced
145	or/142-144	27,810	Advanced
146	exp Cohort Studies/	1,522,637	Advanced
147	exp Prognosis/	1,240,142	Advanced
148	exp Morbidity/	425,952	Advanced
149	exp Mortality/	309,548	Advanced
150	exp survival analysis/	214,369	Advanced
151	exp models, statistical/	311,009	Advanced
152	prognos*.mp,kw.	603,945	Advanced
153	predict*.mp,kw.	1,026,266	Advanced
154	course*.mp,kw.	467,535	Advanced
155	diagnosed.mp,kw.	361,373	Advanced
156	cohort*.mp,kw.	388,862	Advanced
157	death?.mp,kw.	646,834	Advanced
158	or/146-157	4,572,550	Advanced
159	108 and 145 and 158	64	Advanced
160	43 and 145 and 158	122	Advanced
161	159 or 160	184	Advanced
162	limit 161 to yr = “2013-Current”	85	Advanced

^a^Ovid MEDLINE®, 1946–April week 2 2016

## Appendix 2

**Table 4:** Detailed characteristics of included studies

Author	Year	Number of patients	Disease stage	NLR cutoff value	Median age (years)	Breast cancer subtype (%)			Grade (%)		Postmenopausal (%)	Median follow-up (years)
						ER+	HER-2+	Triple negative	Grade 1–2	Grade 3		
Asano et al. [12]	2016	61	Early	3.0	n/a	0	0	100	72	28	36	3.1
Azab et al. [23]	2012	316	Mixed	3.3	n/a	83	17	n/a	70	30	n/a	3.8
Azab et al. [13]	2013	437	Mixed	3.3	64	76	n/a	n/a	n/a	n/a	n/a	5
Bozkurt et al. [24]	2015	85	Early	2.0	n/a	0	0	100	31	69	69	n/a
Dirican et al. [25]	2015	1527	Mixed	4.0	n/a	68	17	n/a	80	20	44	2.5
Forget et al. [10]	2014	720	Early	3.3	n/a	84	9	n/a	61	39	n/a	5.8
Hong et al. [29]	2015	487	Early	1.9	55	67	21	19	73	27	42	4.6
Jia et al. [14]	2015	1570	Early	2.0	47	n/a	22	14	62	38	n/a	6.6
Koh et al. [8]	2014	157	Early	2.3	44	n/a	0	0	80	20	n/a	1.8
Koh et al. [15]	2015	1435	Mixed	5.0	52	55	36	100	56	44	n/a	n/a
Nakano et al. [9]	2015	167	Early	2.5	58	78	18	n/a	80	20	25	7.2^a^
Noh et al. [26]	2013	442	Early	2.5	50	71	29	18	71	29	n/a	5.9
Pistelli et al. [27]	2015	90	Early	3.0	53	0	0	100	10	90	40	4.5
Rimando et al. [28]	2016	461	Mixed	3.8	58	74	n/a	n/a	51	49	n/a	5.1
Yao et al. [11]	2014	608	Early	2.6	53	66	25	16	n/a	n/a	48	3.5

*ER* estrogen receptor, *n/a* not available, *NLR* neutrophil-to-lymphocyte

^a^Mean follow-up

## Abbreviations

CI: Confidence interval
DFS: Disease-free survival
ER: Estrogen receptor
HR: Hazard ratio
NLR: Neutrophil-to-lymphocyte ratio
OS: Overall survival
PFS: Progression-free survival
PR: Progesterone receptor
SE: Standard error
